# Differential Adaptations of Methicillin-Resistant *Staphylococcus aureus* to Serial *In Vitro* Passage in Daptomycin: Evolution of Daptomycin Resistance and Role of Membrane Carotenoid Content and Fluidity

**DOI:** 10.1155/2012/683450

**Published:** 2012-08-16

**Authors:** Nagendra N. Mishra, Aileen Rubio, Cynthia C. Nast, Arnold S. Bayer

**Affiliations:** ^1^Division of Infectious Diseases, Los Angeles Biomedical Research Institute, Harbor-University of California at Los Angeles (UCLA) Medical Center, Torrance, CA 90502, USA; ^2^Cubist Pharmaceuticals, Lexington, MA 02421, USA; ^3^Cedars-Sinai Medical Center, Los Angeles, CA 90048, USA; ^4^David Geffen School of Medicine UCLA, Los Angeles, CA 90024, USA

## Abstract

Previous studies showed serial 20 d *in vitro* passage of MRSA strain MW2 in sublethal daptomycin (DAP) resulted in diverse perturbations in both cell membrane (CM) and cell wall (CW) characteristics, including increased CM rigidity; increased CW thickness; “gain-in-function” single nucleotide polymorphisms (SNPs) in the *mprF* locus (i.e., increased synthesis and translocation of lysyl-phosphatidylglycerol (L-PG)); progressive accumulation of SNPs in *yyc* and *rpo* locus genes; reduced carotenoid production; cross-resistance to innate host defense peptides. The current study was designed to characterize the reproducibility of these phenotypic and genotypic modifications following *in vitro* serial passages of the same parental strain. After a second 20d serial *in vitro* passage of parental MW2, emergence of DAP-R was associated with evolution of several phenotypes closely mirroring previous passage outcomes. However, in contrast to the initial serial passage strain set, we observed (i) only modest increase in L-PG synthesis and no increase in L-PG outer CM translocation; (ii) significantly increased carotenoid synthesis (*P* < 0.05); (iii) a different order of SNP accumulations (*mprF* ≫ *rpoB* ≫ *yycG*); (iv) a different cadre and locations of such SNPs. Thus, MRSA strains are not “pre-programmed” to phenotypically and/or genotypically adapt in an identical manner during induction of DAP resistance.

## 1. Introduction

Invasive *Staphylococcus aureus *infections are rapidly increasing worldwide. The acquisition of multiantibiotic resistances, especially amongst MRSA strains, poses a major problem for clinicians [1–3]. Daptomycin (DAP) has shown great efficacy *in vitro* and *in vivo* against many Gram-positive bacteria including MRSA [4]. DAP has been shown to bind to the bacterial CM, in a calcium-dependent manner, eventually perturbing the CM and dissipating the CM electrochemical gradient, leading to cell death [4, 5]. We and others have identified several genetic loci which correlate to the DAP-resistant (DAP-R) phenotype, including *mprF, vraRS, tag,* and *dltABCD *[6–9]. In these scenarios, either genotypic overexpression and/or phenotypic gains-in-function of these loci were observed, usually featuring single nucleotide polymorphisms (SNPs) [2, 6, 10]. In contrast, in rare cases, other DAP-R *S. aureus* isolates have no identifiable SNPs in any of the above loci [2]. Similarly, many, but not all DAP-R *S. aureus* isolates exhibit a thickened cell wall (CW) phenotype [11]. Thus, these investigations have strongly suggested that the DAP-R phenotype is multifactorial and probably strain-specific.

Friedman et al. [5] previously characterized a set of serially DAP-passaged MRSA isolates (in the MW2 background) for sequential evolution of DAP-R *in vitro* in consort with progressive accumulation of SNPs in *mprF*, *yycG,* and *rpoB/rpoC*. These latter three gene loci encode proteins which are involved in maintaining positive surface charge (MprF), the cell envelope stress response (YycG), and RNA polymerase functions (RpoB/RpoC), respectively. Using this same strain set, we recently reported on the phenotypic correlates of this evolving DAP-R following serial *in vitro* passage in sublethal DAP [6]. We demonstrated distinct changes in a number of phenotypes comparing the parental MW2 strain with the postpassage isolates, including CM fluidity, CM phospholipid profiles, CW thickness, and cross-resistance to host defense cationic peptides from polymorphonuclear leukocytes and platelets [6].

The objective of the present study was to examine the hypothesis that specific *S. aureus* isolates may not be “pre-programmed” in their adaptation to DAP exposures; therefore, such strains may, in fact, evoke distinct and multifactorial mechanisms of response to DAP in order to resist its staphylocidal effect. We, thus, tested the same MRSA parental strain (MW2) that had been repassaged in sublethal DAP following a similar protocol as before [5] and recatalogued key and relevant serial genotypic and phenotypic perturbations. (This work was presented in part at the 113th General Meeting of the American Society for Microbiology, San Francisco, CA; USA, June 16-19, 2012).

*Note.* Although the terminology “daptomycin-nonsusceptibility” is often employed (since there is no officially published CLSI breakpoint), we will use the term “daptomycin-resistance” (DAP-R) for ease of presentation.

## 2. Materials and Methods

### 2.1. Bacterial Strains: Minimum Inhibitory Concentrations (MICs)

We used the same MW2 parental strain as previously reported [6], which then underwent a similar 20 d serial passage protocol in sublethal DAP as described elsewhere [5] (Table 1). For selected investigations (e.g., carotenoid quantifications), the previously DAP-passaged strain set was tested in parallel with the current DAP-passaged strain set, since such assays were not performed in our previous study [6]. DAP, oxacillin (OX), and vancomycin (VAN) MICs were determined by standard *E*-test (AB Biodisk, Devagen, Sweden) on Mueller-Hilton agar (MHA) plates (supplemented with 50 *μ*g/mL calcium chloride for DAP *E*-tests).

### 2.2. Host Defense Peptide (HDP) Susceptibilities

The human neutrophil *α*-defensin-1 (hNP-1) was purchased from Peptides International (Louisville, KY). The hNP-1 killing assay was performed in modified Minimal Essential Media (1% BHI + 10 mM potassium phosphate buffer). A final bacterial inoculum of 10^3^ stationary phase CFU was employed. Two hNP-1 concentrations were used in these assays (10 and 20 *μ*g/mL), representing the highest range of peptide concentrations that did not cause complete killing of the parental MW2 isolate in pilot studies. After 2 h peptide exposure, samples were obtained and quantitatively cultured to evaluate the extent of killing by hNP-1. Final data were expressed as mean (±SD) percent surviving CFU/mL. Since there is no *bona fide* “resistance” breakpoint for HDPs, we utilized the mean percent survival (±SD) to statistically compare the parental strain with the postpassage isolates with increased DAP MICs. A minimum of three experimental runs on separate days was performed.

### 2.3. CM Fluidity

*S. aureus* strains were grown in BHI broth to late stationary phase (18–20 h) at 37°C. CM fluidity was determined by fluorescence polarization spectrofluorometry as detailed elsewhere [2, 6], using the fluorescent probe 1,6-diphenyl-1,3,5-hexatriene (DPH). An inverse relationship exists between polarization indices and the degree of CM order (i.e., lower polarization indices (PI value) equate to greater CM fluidity) [2, 6]. These assays were performed a minimum of six times for each strain on separate days.

### 2.4. Quantification of Carotenoids

The modified protocol of Chamberlain et al. [12] was followed for the quantification of carotenoids. *S. aureus* cells were grown in BHI broth to late stationary phase (18–20 h) at 37°C as above, then harvested and washed and pelleted in PBS by centrifugation. Excess liquid was removed from the final pellets by inversion for at least 2 min and then pellet wet-weight determined. One mL methanol was then added to 0.5 g of *S. aureus* pellet for the extraction of carotenoid. The carotenoid content was determined at 450 nm wavelength, spectrophotometrically [13]. The assay was repeated a minimum of three times for all strains on different days.

### 2.5. CM Phospholipids (PLs) and Amino-PL (L-PG) Asymmetry

The detailed procedures for PL extraction and fluorescamine labeling of outer CM amino-PLs (to determine L-PG translocations) have been previously described in detail [4, 5]. In brief, the major CM PLs of *S. aureus* (phosphatidylglycerol (PG), lysyl-PG (L-PG), and cardiolipin (CL)) were separated by 2D thin-layer chromatography using Silica 60 F254 HPTLC plates (Merck). First-dimension chloroform-methanol 25% ammonium hydroxide (65 : 25 : 6, by volume) in the vertical orientation and second-dimension chloroform : water : methanol : glacial acetic acid : acetone (45 : 4 : 8 : 9 : 16, by volume) in the horizontal orientation were used for the separation of the PLs for further quantitation by phosphate estimation. For quantitative analysis, isolated PL spots were digested at 180°C for 3 h with 0.3 mL 70% perchloric acid and quantified spectrophotometrically at OD_660_ [6, 13, 14].

### 2.6. Fatty Acid Composition

 The extraction of fatty acids was carried out by saponification, methylation, and subsequent conversion into the methyl ester form as previously described [6, 13, 14]. The resulting methyl ester mixtures were separated by an Agilent 5890 dual-tower gas chromatograph. Fatty acids were identified by a microbial identification system, using known standards (Sherlock 4.5; courtesy of Microbial ID Inc., Newark, DE).

### 2.7. Surface Charge

 The relative surface charge was determined by a cytochrome *c* binding assay as described previously [14]. Briefly, overnight culture of *S. aureus* was grown in BHI broth, washed with 20 mM MOPS buffer (pH 7.0) and resuspended in the same buffer at OD_578_ = 1.0. Cells were incubated with 0.5 mg/mL cytochrome *c *for 10 min, and the amount of cytochrome *c *remaining in the supernatant was determined spectrophotometrically at OD_530_ nm. The more unbound cytochrome *c *that was detected in the supernatant, the more relative positive charge on the bacterial surface. Data were expressed as mean (±SD) amount of unbound cytochrome *c*. At least three independent runs were performed on separate days.

### 2.8. Cell Wall (CW) Thickness

CW thickness was measured by transmission electron microscopy as described previously [11]. The mean (±SD) thickness of 100 cells was determined for strains set at a magnification of 190,000 × (JEOL, Model# 100CX, Tokyo, Japan) using digital image capture and morphometric measurement (Advanced Microscopy Techniques v54, Danvers, MA).

### 2.9. Sequencing of *mprF, yycF* and *yycG*, and *rpoB* and *rpoC* SNPs

 Previous studies related to DAP passage of strain MW2 had indicated serial accumulation of SNPs in four gene loci, *acetate coA ligase*, *mprF*, *yycFG,* and *rpoB/rpoC* [5]. To test the reproducibility of these genetic modifications, these loci were resequenced within the new DAP serial passage strain-set. The PCR amplification, sequencing primer sets, and the specific mutation identifications for both strain sets have been previously published [5].

### 2.10. Statistical Analysis

The two-tailed Student *t*-test was used for statistical analysis of all quantitative data. *P* values of ≤0.05 were considered “significant”. 

## 3. Results

### 3.1. MICs

The MICs of DAP, VAN, and OX are listed in Table 1. As noted, the parental MW2 strain (prepassage; CB1118) is DAP-S and VAN-S, but OX-R. Following a 20 d serial passage in sublethal DAP, the DAP MIC progressively rose to 16 *μ*g/mL, reaching the DAP-R range (3 *μ*g/mL) by day 6 of passage. Similarly, the VAN MICs exhibited a progressive rise during passage into the VISA range (3–6 *μ*g/mL). Interestingly, a reduction in OX MICs into the OX-S range was also observed in serially passaged strains, also beginning at day 6 of passage. This event represented the so-called see-saw effect in which progressive rises in DAP-R are accompanied by serial reductions in OX-R profiles [15, 16].

### 3.2. SNPs

 In addition to the previous changes in antibiotic susceptibilities, serial passage in DAP induced sequential accumulation of SNPs over time as compared to the parental strain in the four key target gene loci queried in this investigation, *acetate coA ligase*, *mprF, yycFG,* and *rpoB/rpoC* (Table 1). Thus, in comparing the current serial passage SNP outcomes with those of Friedman et al. [5], the following outcomes ensued: (i) in both the previous and current study, SNPs in *acetate coA ligase* occurred within 1 d of DAP passage; (ii) SNPs in *mprF* became manifest in both studies within the first 5-6 d of DAP passage; and (iii) of interest, although the site of the *mprF* SNP (amino acid position 345) was conserved in both investigations, the specific SNP was not (T345I in the previous study versus T345A in the present study); (iv) as opposed to the previous study in which the next SNP, in *rpoB*, occurred within 14 d of DAP passage, in the current passage strain-set, SNPs in both *yycG* and *rpoB* occurred by this juncture; (v) the location and nature of the SNPs in these latter two loci were also distinctly different in the two investigations; (vi) in the current study, but not in the previous one, by 20 d passage an SNP in *rpoC* was observed.

### 3.3. HDP Susceptibilities

The serially passaged strains showed incremental reductions in killing by hNP-1. By day 6 of passage, the postpassage isolate was already substantially less susceptible to hNP-1 killing as compared to the parental strain (Table 2). By 13 d of DAP passage, with high-level DAP-R already induced (MICs of 8 *μ*g/mL), this same isolate was highly cross-resistant to hNP-1 as compared to the parental strain (*P < *0.05). 

### 3.4. Surface Charge

There were no observable shifts in relative surface charge profiles among the passage strain set, with all strains exhibiting similar patterns of cytochrome *c* binding (data not shown).

### 3.5. PL Composition and Amino-PL Asymmetry

Negatively charged PG was the predominant CM PL in all study strains, ranging from ~78 to 85% of PLs (Table 3). The proportion of the total (inner + outer CM) positively charged PL, L-PG, was modestly increased in two of the early postpassage DAP-R isolates (CB2207 and CB2208) strains, although this was not a durable modification in later postpassage strains. The relative proportion of L-PG which was translocated to the outer CM leaflet did not differ within the strain set. The amount of CL in the strain-set was low and not significantly different among isolates (Table 3). 

### 3.6. Fatty Acid Composition

The composition of fatty acids was similar within the strain set in terms of iso- and anteiso (branched chain) fatty acids (BCFA), as well as straight chain saturated fatty acids (SCFAs) and unsaturated fatty acids (UFAs) (data not shown).

### 3.7. CM Fluidity

There was a notable and significant trend towards increasing CM rigidity over the 20 d DAP passage duration (Table 4). 

### 3.8. Carotenoid CM Content

There was a significant increase in CM carotenoid content beginning early after DAP passage in rough parallel to the progressive increases in DAP MICs (Table 4). In contrast, in the initial DAP-passaged strain-set [6], no increases in carotenoid synthesis were detected over the 20 d passage period (e.g., parental strain OD_450_ = 0.413 ± 0.016 versus 0.351 ± 0.09 and 0.333 ± 0.084, for the 13 and 20 d passage isolates, resp.).

### 3.9. CW Thickness

The DAP-R strains exhibited significantly thicker CWs than the parental strain as shown in Table 4 (*P* < 0.0001).

## 4. Discussion

To expand the knowledge base about potential mechanisms of DAP-R, as well as general staphylococcal responses to cationic peptide-induced stress, we examined the reproducibility of genotypic and phenotypic adaptations to serial *in vitro* DAP exposures in MRSA strain, MW2 (i.e., do specific *S. aureus* strains respond to DAP stress in a predictable and “pre-programmed” manner?). Thus, we took advantage of previously published data which had catalogued both phenotypic and genotypic modifications occurring during serial *in vitro* passage in DAP [5, 6] and compared those to a separate but parallel passage study using the same parental MRSA strain. Phenotypically, we focused on several CM and CW characteristics that have been shown to be perturbed in many DAP-R *S. aureus* strains such as CM order and CW thickness. In addition, we examined cell surface charge and evolution of cross-resistance between DAP and a prototypical anti-*S. aureus* HDP molecule contained within mammalian white blood cells (hNP-1). Genotypically, we concentrated on the identification and order of accumulation of SNPs in four target gene loci which exhibited well-characterized mutations in the earlier passage study: *mprF*, *yycFG*, *rpoB/rpoC*, and *acetate coA ligase* [5].

A number of interesting observations emerged from this study. As expected, there were several definite parallelisms in phenotypic assay outcomes between the current strain set and the prior *in vitro *passaged strain set. Thus, DAP MICs increased sequentially, beginning within the first week of serial DAP exposures. The increases in DAP MICs tracked with incremental and significant increases in cross-R to the prototypical host defense peptide, hNP-1, increased CW thickness and reduced CM fluidity. In addition, and as seen in the prior passage study, there was no significant pattern of alterations in surface charge during DAP passage *in vitro*. Moreover, the profile of CM fatty acids neither differed between the passage sets nor within each passage set.

There were two major phenotypic differences detected in comparing the original with the present DAP-passaged strain-sets: (i) CM carotenoid content and (ii) CM phospholipid (PL) composition. In the prior passage study, there was a trend towards reduced CM carotenoid content over the 20 d passage period. In contrast, there was a clear, progressive, and significant increase in carotenoid synthesis amongst the current passage strain-set observed. This phenotype fits quite well with the sequential reductions in CM fluidity observed in this latter strain set, since CM carotenoids can be major contributors to the overall CM “scaffolding” and its rigidity characteristics [12, 13]. Further, our recent studies on forced carotenoid overproduction in *S. aureus* via multicopy plasmid linked this phenotype with both DAP MIC increases and cross-resistances to key host defense peptides [2]. 

In terms of differences in CM PL compositions between the two strain-sets, in the previous passage strain-set, there was a significant increase in total L-PG synthesized and in the proportion of L-PG flipped to the outer CM as compared to the parental strain (i.e., ~25% of total PLs, with ~33% flipped versus ~12% of total PLs, and ~10% flipped, resp.) [6]. These differences appeared to correlate with identification of an SNP within the bifunctional domain of the *mprF* locus detected in the post-DAP passage strain [6]. 

In contrast, in the current strain-set, there was only a modest increase in total L-PG and no substantive increases in proportion of L-PG flipped to the outer CM in comparing the parental with the day 20 passage isolate. This lack of effect on L-PG synthesis or translocation occurred despite acquisition of a SNP within *mprF*. Of interest, we have previously documented a similar phenomenon in a different clinically derived DAP-R isolate, in which an *mprF* SNP was not accompanied by either increased expression levels or phenotypic gains-in-function, *viz-a-viz* L-PG synthesis or flipping [7]. 

Of note, there were also major differences between the original passage strain-set and the current set in the profile of SNPs accumulated within the key target gene loci queried, both in terms of the sequential acquisition of SNPs and the specific amino acid substitutions observed. Thus, despite mutations first occurring in the *acetate coA ligase* gene, then followed by mutations in *mprF* in both passage series, the specific *mprF* SNP amino acid substitution differed between strain sets (i.e., *mprF*_T345I_ versus *mprF*_T345A_, resp.). Similarly, the location and amino acid substitutions of SNPs within the *rpoB* and *yycG* loci differed between the first set (*rpoB*_I935S_; *yycG*_S221P_) and current set (*rpoB*_A1086V_; *yycG*_R263C_). Additionally, the order in which SNPs accumulated was different; in comparing the previous and current passage strain sets differed (*mprF*_T345I_ ≫ *rpoB*_I935S_ ≫ *yycG*_S221P_ versus *mprF*_T345A_ ≫ *yycG*_R263C_ ≫ *rpoB*_A1086V_ ≫ *rpoC*_Q961 K_, resp.). As detailed before, in the previous study (but not in the present investigation), we observed evidence of *mprF* phenotypic gains-in-function (i.e., increased L-PG synthesis and flipping), even though nearly identical SNPs were acquired by both strain-sets during DAP passage [6]. This speaks to either (i) synonymous SNPs, (ii) subtle, but important, functional differences in the MprF protein depending on amino acid structure, or (iii) involvement of gene loci or networks outside of *mprF* which influence PL phenotypes. In the same way, the phenotypic impacts of SNP differences in *yycG* and/or *rpoB/rpoC*, as well as the temporal order in which they are accumulated, remain to be determined. Of interest, two recent studies have examined isogenic DAP-S/DAP-R strain pairs for their comparative genomic profiles. Peleg et al. [17] demonstrated by whole genome sequencing that DAP-R *S. aureus* strains exhibited mutations in genes responsible for phospholipid biosynthesis, especially, *mprF, cls2* (involved in cardiolipin biosynthesis), and *pgsA *(involved in PG synthesis). Similarly, Boyle-Vavra et al. compared the genome sequences of a clinical DAP-R MRSA strain from a patient exhibiting treatment failure to DAP with its initial DAP-S isolate; they also found a point mutation in *mprF *[18].

In summary, it seems reasonable to conclude that an individual *S. aureus* strain can respond to DAP exposures by any number of adaptive mechanisms at both genotypic and/or phenotypic levels. Clearly, specific *S. aureus* strains are not “pre-programmed” to resist DAP-induced killing by any single pathway. Of these DAP adaptations, it appears that alterations in CM order, increases in CW thickness, and perturbations in CM PL content are among the major response mechanisms. 

## Figures and Tables

**Table 1:** List of strains and MICs.

Strains	Length of serial passage (days)	Gene mutation(s) ^b^	DAP (*μ*g/mL)	VAN (*μ*g/mL)	OX (*μ*g/mL)
Parental CB1118 (MW2)		—	1	2	24
CB2206	1	*acetate coA ligase* ^ a^	1.5	2	24
CB2207	6	*acetate coA ligase*; *mprF*_T345A_	3	3	2
CB2208	9	*acetate coA ligase; m* *pr* *F*_T345A_; *yycG*_R263C_	4	4	2
CB2209	13	*acetate coA ligase*; *mprF*_T345A_; *yycG*_R263C_ *rpoB*_A1086V_	8	6	2
CB2210	20	*acetate coA ligase*; *mprF*_T345A_; *yycG*_R263C_; *rpoB*_A1086V_; *rpoC*_Q961K_	16	6	2

^a^Noncoding region of coenzyme A.

^b^The data have been previously published (s).

**Table 2:** *In vitro* susceptibility profiles to host defense peptides (HDPs).

Strains	hNP-1 (10 *μ*g/mL)	hNP-1 (20 *μ*g/mL)
CB1118	25 ± 14	12 ± 13
CB2206	20 ± 12	9 ± 9
CB2207	46 ± 13^∗+^	15 ± 7
CB2208	68 ± 10^∗^	55 ± 32^∗+^
CB2209	102 ± 19^∗^	101 ± 26^∗^
CB2210	99 ± 16^∗^	94 ± 15^∗^

^∗^ *P* value < 0.05 versus parental strain; ^∗+^ *P* value < 0.08 versus parental strain.

**Table 3:** Cell membrane (CM) phospholipid composition and asymmetry of L-PG.

% of total phospholipid (mean ± SD)					
Strains	Inner CM L-PG	Outer CM L-PG	Total CM L-PG	Total CM PG	Total CM CL
CB1118	11.69 ± 0.90	1.57 ± 0.41	13.26 ± 0.49	84.85 ± 1.74	1.89 ± 1.25
CB2206	13.30 ± 0.91	1.94 ± 0.87	15.24 ± 0.04	79.38 ± 1.26	5.38 ± 1.30
CB2207	18.94 ± 5.00^∗^	2.02 ± 0.27	20.97 ± 4.73^∗^	75.33 ± 5.55	2.90 ± 1.96
CB2208	18.69 ± 4.42^∗^	2.17 ± 0.53	20.87 ± 3.89^∗^	77.81 ± 2.97	4.22 ± 3.17
CB2209	13.04 ± 2.56	2.24 ± 0.84	15.29 ± 1.72	83.06 ± 1.66	1.65 ± 0.07
CB2210	14.27 ± 0.80	2.72 ± 0.97	17.00 ± 1.77^∗^	81.45 ± 2.56	1.56 ± 0.79

^∗^ *P*-value < 0.05 versus parental strain.

**Table 4:** Quantification of CM carotenoid pigment, CM fluidity and CW thickness.

Strains	Carotenoids (OD_450_)	CM fluidity (PI-value)	CW thickness (nm)
CB1118	0.49 ± 0.003	0.279 ± 0.0255	26.42 ± 3.9
CB2206	0.50 ± 0.032	0.321 ± 0.0285^∗^	25.53 ± 3.1
CB2207	0.63 ± 0.099	0.304 ± 0.0165	28.91 ± 3.2^∗∗^
CB2208	0.67 ± 0.054^∗^	0.365 ± 0.0398^∗^	32.05 ± 4.4^∗∗^
CB2209	0.65 ± 0.064^∗^	0.357 ± 0.0407^∗^	33.94 ± 4.2^∗∗^
CB2210	0.75 ± 0.014^∗^	0.392 ± 0.0552^∗^	42.24 ± 6.2^∗∗^

^∗^ *P* value < 0.05 versus parental strain; ^∗∗^ *P* value < 0.0001 versus parental strain.

A lower PI value equates to a greater extent of CM fluidity.
